# Association Between Uterine Volume and In Vitro Fertilization (IVF) Reproductive Outcomes of Infertile Patients with Adenomyosis

**DOI:** 10.1007/s43032-023-01210-2

**Published:** 2023-05-15

**Authors:** Wen Zhang, Ningning Pan, Bing Han, Xiaoxue Li, Rong Li, Caihong Ma, Jie Qiao

**Affiliations:** 1 Center for Reproductive Medicine, Department of Obstetrics and Gynecology, Peking University Third Hospital, Beijing, 100191 China; 2 National Clinical Research Center for Obstetrics and Gynecology (Peking University Third Hospital), Beijing, 100191 China; 3 Key Laboratory of Assisted Reproduction, Ministry of Education (Peking University), Beijing, 100191 China; 4 Beijing Key Laboratory of Reproductive Endocrinology and Assisted Reproductive Technology, Beijing, 100191 China; 5 Research Units of Comprehensive Diagnosis and Treatment of Oocyte Maturation Arrest, Chinese Academy of Medical Sciences, Beijing, 100191 China

**Keywords:** Adenomyosis, Infertility, Uterine volume, In vitro fertilization

## Abstract

To explore the association between uterine volume and in vitro fertilization (IVF) reproductive outcomes of infertile patients with adenomyosis, we performed a retrospective cohort study of infertile patients with adenomyosis who underwent IVF from January 2009 to December 2019 in our clinical center. Patients were divided into five groups according to the uterine volume before the IVF cycle. A line graph was drawn to demonstrate the linear trend of IVF reproductive outcomes with uterine volume. Univariate and multivariate analyses were used to explore the association between uterine volume of adenomyosis patients and IVF reproductive outcomes in first fresh embryo transfer (ET) cycle, first frozen-thawed embryo transfer (FET) cycle, and per ET cycle. Kaplan–Meier curves and Cox regression were conducted to evaluate the association between uterine volume and cumulative live birth. A total of 1155 infertile patients with adenomyosis were included. Clinical pregnancy rate showed no significant correlation with uterine volume in first fresh ET cycle, first FET cycle, and per ET cycle; miscarriage rate showed an upward trend with uterine volume increasement, in which the uterine volume turning point was 8 weeks of gestation; live birth rate showed a downward trend with turning point of 10 weeks of gestation. Subsequently, patients were divided into two groups (uterine volume ≤ 8 weeks of gestation vs. uterine volume > 8 weeks of gestation). Univariate and multivariate analyses showed that patients with a uterus larger than 8 weeks of gestation had a higher miscarriage rate and a lower live birth rate in all ET cycles. Kaplan–Meier curves and Cox regression demonstrated lower cumulative live birth rate in patients with a uterine volume larger than 8 weeks of gestation. IVF reproductive outcome gets worse as uterine volume increases in infertile patients with adenomyosis. Adenomyosis patients with a uterus larger than 8 weeks of gestation had a higher miscarriage rate and a lower live birth rate.

**Supplementary Information:**

The online version contains supplementary material available at 10.1007/s43032-023-01210-2.

## Introduction

Adenomyosis is a common gynecological disease in women of reproductive age which is characterized by endometrial glands and stroma in the normal myometrium, accompanied by hypertrophy of the surrounding myometrial smooth muscle cells [1]. The main clinical manifestations of adenomyosis include dysmenorrhea, abnormal uterine bleeding, and infertility.

The results of several studies showed that the clinical pregnancy rate and live birth rate of in vitro fertilization-embryo transfer (IVF-ET) decreased in infertile patients with adenomyosis [2–4]. However, there is currently a lack of appropriate evaluation indicators to evaluate the severity degree of adenomyosis, as well as the lack of prognostic indicators for IVF reproductive outcomes in infertile patients with adenomyosis. For adenomyosis, ectopic endometrium grows diffusely in the myometrium, resulting in the enlargement of the uterus, obviously increasing anterior and posterior diameter and the uterus might often be a spherical shape. Ultrasound examination showed that the uterus was enlarged, the myometrium was thickened, and the lesion site is iso-echo or echo enhancement, with punctate hypoecho, and there is no obvious boundary between the lesion and the surrounding. Mavrelos D et al. showed that more accumulation of adenomyosis features on ultrasound was associated with a lower clinical pregnancy rate after IVF in infertile women with adenomyosis [5]. Many studies have suggested that pretreatment with a gonadotrophin-releasing hormone agonist (GnRH-a) could reduce the volume of the uterus and thus improve the clinical pregnancy rate of infertile patients with adenomyosis [6, 7]. Previous studies by our research group have demonstrated the impact of uterine volume on reproductive outcomes in adenomyosis patients. We included a total of 158 infertile patients with adenomyosis, and the uterine volume exceeded 100 cm^3^ before frozen-thawed embryo transfer (FET) had an increased risk of miscarriage [3, 8, 9].

The above findings suggest that the uterine volume of adenomyosis is closely related to IVF reproductive outcomes. A consensus of experts in China recommended natural pregnancy after GnRH-a pretreatment for adenomyosis-associated infertile patients with a uterine volume smaller than 12 weeks of gestation and recommended IVF until uterine volume larger than 12 weeks of gestation [10]. However, we doubted that adenomyosis patients will miss the best opportunity for IVF if IVF was recommended until the uterine volume was larger than 12 weeks of gestation. The association between different uterine volumes of adenomyosis patients and IVF reproductive outcomes remains unknown. Therefore, our research team included ten years of IVF-ET clinical data of adenomyosis-associated infertility patients and detailly recorded the uterine volume of each patient to explore the association between uterine volume and IVF reproductive outcomes.

## Methods

### Study Design and Patients

This was a retrospective cohort study of infertile patients with adenomyosis who underwent IVF-ET at the Reproductive Center of the Peking University Third Hospital from January 2009 to December 2019. This research was approved by the Ethical Review Committee of Peking University Third Hospital (No. LM2021243). The individual consent for this retrospective analysis was waived.

The inclusion criteria were as follows: Patients were diagnosed as adenomyosis by transvaginal ultrasound scans (TVS) [11], and the TVS were performed by two experienced sonographers; aged ≤ 45 years old at the first outpatient visit to our Reproductive Center; with the regular menstrual cycle. The criteria for sonographic diagnosis of adenomyosis are with 2 or more of the following: heterogeneous myometrial texture with the presence of a globular asymmetric uterus, thickening of the anterior and posterior myometrial wall, and irregular cystic areas within the myometrium [12]. Exclusion criteria were listed as follows: patients with intrauterine adhesion, uterine malformation, submucosal leiomyoma, or ≥ 5.0 cm in diameter leiomyoma; hydrosalpinx and systemic diseases; and patients with other endocrine severe diseases, immune diseases, tumors, and abnormal chromosomes in either partner.

### Uterine Volume Measurement and Corresponding Gestational Weeks

Each adenomyosis patient underwent gynecological ultrasound prior to the initiation of IVF-ET. The uterine volume was calculated by using a geometric formula for a prolate ellipsoid volume: long diameter × width diameter × anteroposterior diameter × π/6 [13]. Uterine volume and the corresponding gestational weeks were listed as follows: Uterine volume ≤ 56 cm^3^ was corresponding to ≤ 4 weeks of gestation, 56–90 cm^3^ was corresponding to 4–6 weeks of gestation, 90–130 cm^3^ was corresponding to 6–8 weeks of gestation, 130–180 cm^3^ was corresponding to 8–10 weeks of gestation, and > 180 cm^3^ was corresponding to > 10 weeks of gestation [14] (see Supplementary Table 1).

### IVF Protocol and Embryo Transfer

Different controlled ovarian hyperstimulation (COH) protocols were administrated for adenomyosis-associated infertile patients, such as GnRH-a ultralong protocol, GnRH-a long protocol, GnRH-antagonist protocol, and minimal ovarian stimulation protocol [15]. Either recombinant follicle-stimulating hormone (rFSH) or human menopausal gonadotrophins (hMG) were used. Standard methods for oocyte retrieval and fertilization with conventional IVF were used. The quality of embryos was evaluated according to the Istanbul Consensus Workshop on Embryo Assessment criteria [16]. Blastocysts were evaluated according to the Gardner morphological grading system. Embryos/blastocysts transfer was performed on day 3/day 5 in a fresh cycle. Other embryos/blastocysts were vitrificated for cryopreservation. Vaginal and/or intramuscular/oral progesterone were given as luteal support.

### Frozen-thawed Embryo Transfer

GnRH-a pretreatment before FET was determined based on the experience of clinicians and the needs of patients. Patients with GnRH-a pretreatment were injected subcutaneously with long-acting GnRH-a (triptorelin acetate for injection, Ipsen, French, 3.75 mg) for 1–6 months or more, starting from the 1st day to the 5th day of menstruation, once per 28–35 days. Endometrial preparation was started 28 days after the last GnRH-a injection with daily estradiol 4–6 mg, and progesterone was added when the thickness of the endometrium reached 8 mm. After 5–7 days of progesterone treatment, one or two embryos were transferred into the uterus. A natural cycle was applied for patients without GnRH-a pretreatment.

### Clinical Data and Definitions

The basic characteristics of the participants, such as age, body mass index (BMI), infertility type, infertility duration, gravidy, parity times, basal FSH, anti-Müllerian hormone (AMH), uterine volume before IVF cycle, COH protocol, endometrial thickness, number of embryos transferred, and transferred embryo type (cleavage embryo/blastocyst), were evaluated. Clinical pregnancy denoted evidence of at least one intrauterine gestational sac observed by ultrasonography 30 days after embryo transfer. Miscarriage was defined as the presence of an intrauterine gestational sac but no subsequent live birth after 24 weeks of gestation. Live birth was defined as the delivery of a live baby after 24 weeks of gestation.

### Statistical Analysis

Characteristics were presented as mean ± standard deviation (SD) or median (interquartile range, IQR) for continuous variables and percentages for categorical variables. Comparisons between ratios were performed using the chi-square test or Fisher’s exact test. Continuous variables were analyzed by T-tests or nonparametric tests. A line graph was drawn to explore the linear trend of IVF reproductive outcomes with uterine volume. Logistic regression models were used to estimate the effect of uterine volume on reproductive outcomes. Kaplan–Meier (KM) curves were made to compare the cumulative live birth rate between different groups. Since the number of patients with ≥ 5 embryo transfer cycles was small, we analyzed the cumulative live birth rate of the patients during the first 4 embryo transfer cycles. Furthermore, multivariate Cox regression was conducted to evaluate the effect of uterine volume on the cumulative live birth rate. *P* < 0.05 was considered statistically significant. Analysis was performed using the Statistical Package for Social Sciences (SPSS), version 25.0 (IBM, Armonk, New York, USA).

## Results

### Baseline Characteristics of Infertile Patients with Adenomyosis

A total of 1155 infertile patients with adenomyosis were included in this study, and they were divided into five groups according to their uterine volume (presenting with corresponding gestational week), as shown in Supplementary Table 1.

### Baseline Characteristics and Reproductive Outcomes of Adenomyosis-associated Infertile Patients with Different Uterine Volume

Patients’ age gradually increased as uterine volume increased (age in each group was 33.1 years, 34.7 years, 35.0 years, 35.2 years, and 35.7 years, respectively), as did BMI (BMI in each group was 22.4 kg/m^2^, 23.1 kg/m^2^, 23.2 kg/m^2^, 23.7 kg/m^2^, and 23.9 kg/m^2^, respectively), with *P* values of 0.000 and 0.002, respectively. Infertility type, infertility duration, gravidy, basal FSH, and AMH were not statistically significant among the groups.

In order to fully explore the reproductive outcomes in adenomyosis patients with different uterine volumes, our study analyzed from three perspectives: first fresh ET cycle, first FET cycle, and per ET cycle, as shown in Supplementary Table 2. The results are listed as follows: **①** Clinical pregnancy rate showed no statistical differences in first fresh ET cycle, first FET cycle, and per ET cycle with *p* values of 0.143, 0.754, and 0.076, respectively. **②** Miscarriage rate showed an upward trend with the increase of uterine volume, and *P* values in first fresh ET cycle, first FET cycle, and per ET cycle were 0.057, 0.032, and 0.002, respectively. **③** Live birth rate showed a downward trend with the increase in uterine volume, and *P* values were 0.022, 0.100, and 0.001, respectively. *P* values of miscarriage rate and live birth rate in some comparisons showed close to 0.05 but still larger than 0.05 maybe because of the relatively small sample size in each subgroup.

**Fig. 1:**
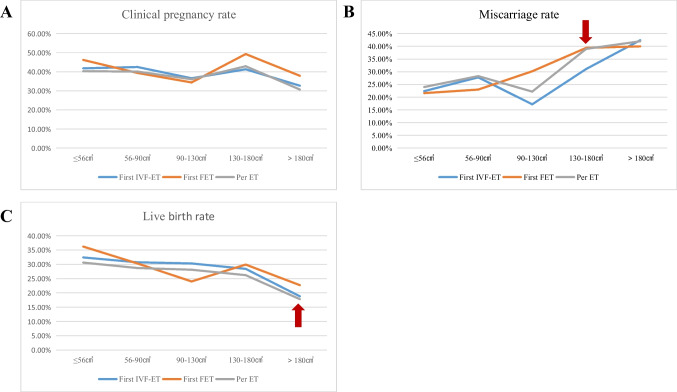
Line graph for reproductive outcomes of adenomyosis patients with different uterine volumes. (**A**) Clinical pregnancy rate varied with uterine volume. (**B**) The miscarriage rate showed an upward trend with uterine volume increasement, and the uterine volume turning point (red arrow) was 8 weeks of gestation. (**C**) Live birth rate showed a downward trend with a turning point (red arrow) of 10 weeks of gestation

What is more, we identified uterine volume turning points for worse reproductive outcomes (i.e., high miscarriage rate and low live birth rate) in infertile patients with adenomyosis. In terms of clinical pregnancy rate, our study did not find a significant uterine volume turning point for a worse clinical pregnancy rate (Fig. 1A). As seen in Supplementary Table 2 and Fig. 1, **①** uterine volume at 8 weeks of gestation (130cm^3^) was the turning point for higher miscarriage rate, i.e., the miscarriage rate increased significantly in adenomyosis-associated infertile patients with uterine volume > 8 weeks of gestation (Fig. 1B). **②** Uterine volume at 10 weeks of gestation (180cm^3^) was the turning point for lower live birth rate, i.e., live birth rate decreased significantly in adenomyosis-associated infertile patients with uterine volume > 10 weeks of gestation (Fig. 1C).

Considering that the miscarriage rate had significantly increased when uterine volume exceeds 8 weeks of gestation, we preliminary considered that uterine volume at 8 weeks of gestation was a watershed for IVF reproductive outcomes in adenomyosis-associated infertile patients, i.e., reproductive outcomes were significantly worse in adenomyosis patients with uterine volume > 8 weeks of gestation, which was further verified by subsequent analysis.

### Reproductive Outcomes of Adenomyosis-associated Infertile Patients with Uterine Volume Grouped by 8 Weeks of Gestation

**Fig. 2:**
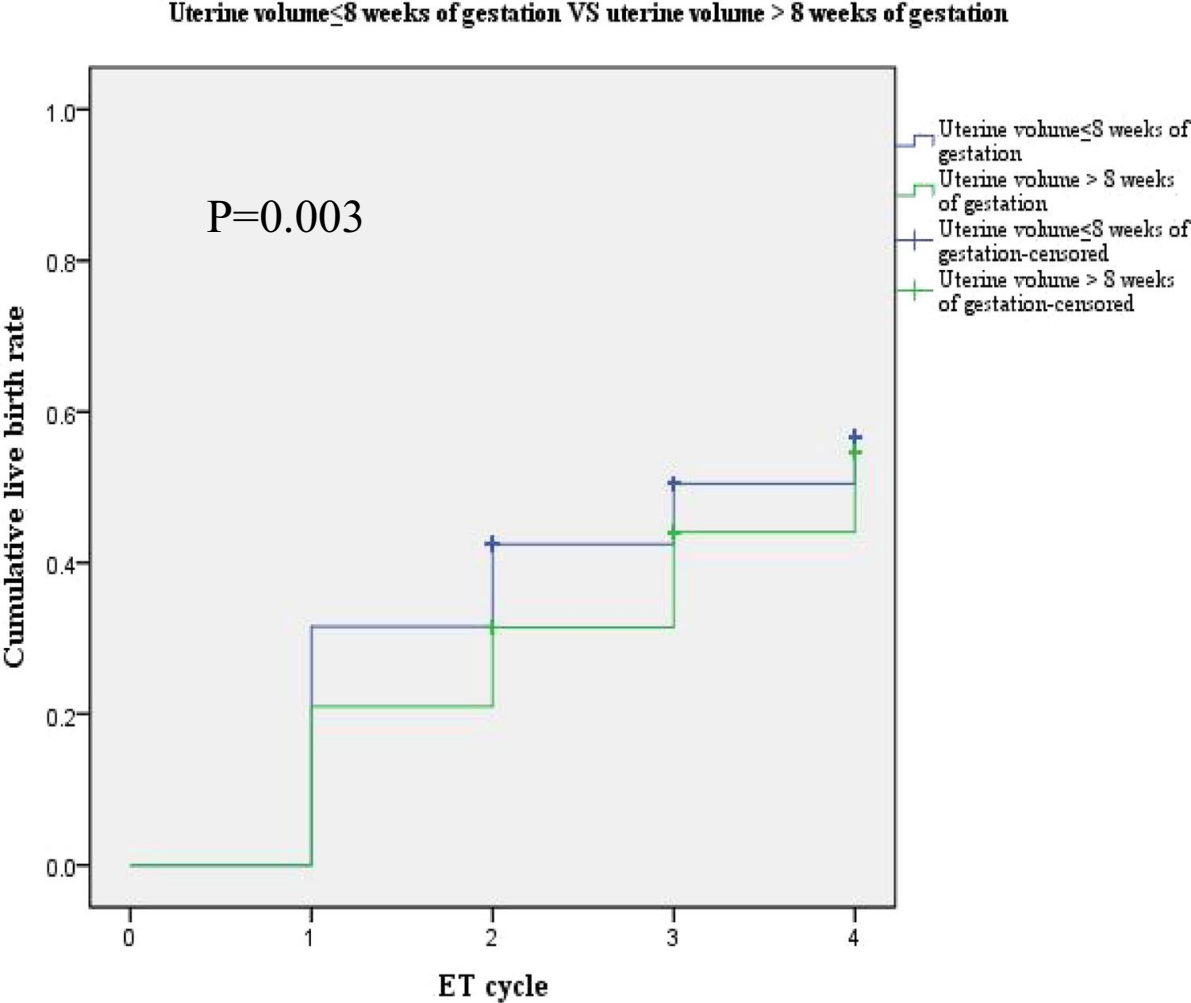
Kaplan–Meier curves comparing cumulative live birth rate between two groups. The cumulative live birth rate significantly decreased in adenomyosis patients with uterine volume > 8 weeks of gestation, with a *p* value of 0.003

We divided the infertile patients with adenomyosis into two groups (uterine volume ≤ 8 weeks of gestation vs. uterine volume > 8 weeks of gestation) and compared reproductive outcomes between the two groups using univariate and multivariate analyses (see Tables 1 and 2), as well as comparing the cumulative live birth rates using KM curves (see Fig. 2) and multivariate Cox regression (Table 2).

**Table 1:** Reproductive outcomes of adenomyosis-associated infertile patients with uterine volume grouped by 8 weeks of gestation – univariate analysis

	Grouped by 8 weeks of gestation, *n* = 1155		
Uterine volume(cm^3^)	≤ 130	> 130	*P*
Number	891	264	
Age (years), mean ± SD	34.4 ± 4.3	35.4 ± 4.1	0.001
BMI (kg/m2), mean ± SD	23.0 ± 3.7	23.8 ± 3.9	0.001
Infertility type, %			0.381
Primary infertility	496/891 (55.7%)	155/264 (58.7%)	
Secondary infertility	395/891 (44.3%)	109/264 (41.3%)	
Infertility duration (years), median (IQR)	3.0 (2.0, 5.0)	4.0 (2.0, 5.0)	0.420
Gravidy, median (IQR)	1.0 (0.0, 2.0)	1.0 (0.0, 2.0)	0.487
Parity times, median (IQR)	0.0 (0.0 0.0)	0.0 (0.0 0.0)	0.376
Basal FSH (mIU/ml), median (IQR)	6.6 (5.3, 8.3)	6.6 (4.9, 8.1)	0.092
AMH (ng/ml), median (IQR)	2.0 (1.0, 3.3)	2.4 (1.3, 3.9)	0.249
	First fresh ET cycle, *n* = 943		
Number	747	196	
COH protocol, %			0.000
GnRH-a ultralong protocol	397/747 (53.1%)	163/196 (83.2%)	
GnRH-a long protocol	144/747 (19.3%)	13/196 (6.6%)	
Other protocols	206/747 (27.6%)	20/196 (10.2%)	
Gn dose (IU), median (IQR)	3300.0 (2400.0, 4350.0)	3525.0 (2837.5, 4650.0)	0.012
Gn days (day), mean ± SD	11.8 ± 2.4	11.7 ± 2.5	0.702
Transferred embryo, %			0.098
Cleavage embryo	715/747 (95.7%)	182/196 (92.9%)	
Blastocyst	32/747 (4.3%)	14/196 (7.1%)	
No. of embryos transferred, mean ± SD	1.9 ± 0.5	1.9 ± 0.5	0.619
Endometrial thickness (mm), mean ± SD	10.7 ± 1.8	10.5 ± 1.9	0.155
Clinical pregnancy rate, %	305/747 (40.8%)	69/196 (35.2%)	0.152
Miscarriage rate, %	72/305 (23.6%)	25/69 (36.2%)	0.031
Live birth rate, %	233/747 (31.2%)	44/196 (22.4%)	0.017
	First FET cycle, *n* = 493		
Number	360	133	
GnRHa pretreatment, %	102/360 (28.3%)	108/133 (81.2%)	0.000
Transferred embryo, %			0.549
Cleavage embryo	157/360 (43.6%)	54/133 (40.6%)	
Blastocyst	203/360 (56.4%)	79/133 (59.4%)	
No. of embryos transferred, mean ± SD	1.5 ± 0.6	1.5 ± 0.6	0.367
Endometrial thickness (mm), mean ± SD	10.0 ± 1.8	9.7 ± 1.5	0.170
Clinical pregnancy rate, %	141/360 (39.2%)	58/133 (43.6%)	0.372
Miscarriage rate, %	35/141 (24.8%)	23/58 (39.7%)	0.036
Live birth rate, %	106/360 (29.4%)	35/133 (26.3%)	0.495
	Per ET cycle, *n* = 1876		
Number	1418	458	
Transferred embryo, %			0.009
Cleavage embryo	1063/1418 (75.0%)	315/458 (68.8%)	
Blastocyst	355/1418 (25.0%)	143/458 (31.2%)	
No. of embryos transferred, mean ± SD	1.8 ± 0.5	1.7 ± 0.6	0.101
Endometrial thickness (mm), mean ± SD	10.4 ± 1.8	10.1 ± 1.8	0.003
Clinical pregnancy rate, %	549/1418 (38.7%)	169/458 (36.9%)	0.487
Miscarriage rate, %	139/549 (25.3%)	68/169 (40.2%)	0.000
Live birth rate, %	410/1418 (28.9%)	101/458 (22.1%)	0.004

*BMI*, body mass index; *SD*, standard deviation; *IQR*, interquartile range; *COH*, controlled ovarian hyperstimulation; *Gn*, gonadotropin

**Table 2:** Reproductive outcomes of adenomyosis-associated infertile patients with uterine volume grouped by 8 weeks of gestation – multivariate logistic analysis/multivariate Cox regression

	OR	95%CI	*P*^#^
	First fresh ET cycle		
Clinical pregnancy rate	0.746	0.526, 1.057	0.099
Miscarriage rate	1.807	1.035, 3.157	0.038
Live birth rate	0.637	0.432, 0.938	0.022
	First FET cycle		
Clinical pregnancy rate	1.349	0.837, 2.174	0.219
Miscarriage rate	2.463	1.104, 5.494	0.028
Live birth rate	0.865	0.510, 1.467	0.591
	Per ET cycle		
Clinical pregnancy rate	1.002	0.798, 1.257	0.989
Miscarriage rate	1.862	1.278, 2.711	0.001
Live birth rate	0.753	0.580, 0.977	0.033
	Cumulative cycle		
Cumulative live birth rate	0.794	0.634, 0.995	0.045

^#^Multivariate logistic analysis adjusting age, BMI, embryos/blastocysts, and COH protocol/GnRHa pretreatment before FET in first IVF-ET cycle, first FET cycle, and per ET cycle; multivariate Cox regression adjusting age, BMI in the cumulative cycle

*OR*, odds ratio; *CI*, confidence interval

### Reproductive Outcomes Between Groups – Univariate and Multivariate Analyses

Adenomyosis patients with uterine volume > 8 weeks of gestation had higher age (34.4 ± 4.3 vs. 35.4 ± 4.1, *P* = 0.001) and BMI (23.0 ± 3.7 vs. 23.8 ± 3.9, *P* = 0.001). Infertility type, infertility duration, gravidy, parity times, basal FSH, and AMH were not statistically significantly different between the two groups (see Table 1).

Similarly, we analyzed the reproductive outcomes of patients from the first fresh ET cycle, first FET cycle, and per ET cycle (see Table 1). **①** In terms of the first fresh ET cycle, there was no statistically significant difference in clinical pregnancy rate between the two groups (40.8% vs. 35.2%, *P* = 0.152); however, the miscarriage rate significantly increased (23.6% vs. 36.2%, *P* = 0.031) and live birth rate significantly decreased (31.2% vs. 22.4%, *P* = 0.017) in adenomyosis patient with uterine volume > 8 weeks of gestation (see Table 1). **②** In the first FET cycle, the miscarriage rate was higher in adenomyosis patients with uterine volume > 8 weeks of gestation (24.8% vs. 39.7%, *P* = 0.036). **③** In per ET cycle, the clinical pregnancy rate was not statistically different between the two groups (38.7% vs. 36.9%, *P* = 0.487), but the miscarriage rate significantly increased (25.3% vs. 40.2%, *P* = 0.000) and live birth rate significantly decreased (28.9% vs. 22.1%, *P* = 0.004) in adenomyosis patient with uterine volume > 8 weeks of gestation.

Multivariate analysis showed the same conclusions after correcting for age, BMI, embryos/blastocysts, and COH protocol/GnRH-a pretreatment before FET (see Table 2).

### Cumulative Live Birth Rate Between Groups – Univariate and Multivariate Analyses

The KM curves showed that cumulative live birth rate significantly decreased in adenomyosis patients with uterine volume > 8 weeks of gestation, with *p* values of 0.003 (see Fig. 2). Multivariate Cox regression adjusted age and BMI and showed the same results, with *p* values of 0.045 (see Table 2).

## Discussion

This study analyzed the IVF reproductive outcomes of adenomyosis-associated infertile patients with different uterine volumes and found that adenomyosis patients with a uterus larger than 8 weeks of gestation had a higher rate of miscarriage and a lower rate of live birth. In addition, our study confirmed this finding from four perspectives (first fresh ET cycle, first FET cycle, per ET cycle, and cumulative live birth rate).

It is widely accepted that adenomyosis could affect IVF reproductive outcomes. Younes G conducted a meta-analysis and found that implantation, clinical pregnancy, ongoing pregnancy, and live birth were significantly lower and the miscarriage rate was higher in adenomyosis patients than in controls [17]. Zhang XP also found that the early miscarriage rate in the adenomyosis group was significantly higher than that in the control group, and the live birth rate was lower [18]. Both of the above studies confirmed the effect of adenomyosis on IVF reproductive outcomes; however, it is unclear whether different types of adenomyosis affect IVF reproductive outcomes to different degrees. The relationship between infertility and clinical subtypes of adenomyosis has also been gradually concerned. Clinical subtypes of adenomyosis included focal/diffuse type and internal/external type. Focal adenomyosis (including adenomyoma) is classified when typical ultrasonographic adenomyotic signs are circumscribed in aggregates and surrounded by normal myometrium. Diffuse adenomyosis is classified when typical alterations at TVS spread throughout the myometrium [19]. Internal adenomyosis was defined as a junctional zone(JZ)max of at least 12 mm and the ratio of the JZmax to the myometrial thickness > 40%. External adenomyosis was defined as an adenomyosis lesion located in the outer shell of the uterus, separated from the JZ, which remained intact and with preserved healthy muscular structures between the adenomyosis and the JZ [20]. Bourdon M conducted a single-center cross-sectional study and found that the presence of focal adenomyosis of the outer myometrium (diagnosed by magnetic resonance imaging) was associated with primary infertility; however, diffuse adenomyosis was not found to be associated with infertility [21]. What is more, they also explored the relationship between internal/external adenomyosis and primary infertility and found that external adenomyosis has a higher proportion of primary infertility than internal adenomyosis [20]. However, the relationship between clinical subtypes of adenomyosis and IVF reproductive outcomes is unclear, and whether there are clinical indicators that could predict IVF reproductive outcomes that deserve to be further explored.

There is a lack of effective predictors of reproductive outcomes in adenomyosis-associated infertile patients undergoing IVF. Adenomyosis is often accompanied by an increase in uterine volume, and as the lesions accumulate, the uterine volume also increases gradually. Uterine volume may play an important role in predicting IVF reproductive outcomes in adenomyosis. Our research group has previously conducted a series of studies on uterine volume and has proven that uterine volume of adenomyosis had adverse effects on IVF-ET and FET reproductive outcomes, and it was recommended to reduce uterine volume as much as possible before embryo transfer [8, 22]. In addition to the consideration of adenomyosis uterine volume before ET, we have always encountered the following question in clinical practice: How about the clinical pregnancy rate, miscarriage rate, live birth rate, and cumulative live birth rate of adenomyosis patients undergoing IVF with different uterine volumes? Therefore, this study addressed the clinical issue by describing the reproductive outcomes of IVF in adenomyosis-associated infertile patients with different uterine volumes before the IVF cycle. What is more, we identified a turning point (uterine volume at 8 weeks of gestation) for worse reproductive outcomes, i.e., adenomyosis patients with a uterus larger than 8 weeks of gestation had a higher miscarriage rate and a lower live birth rate.

The reason why uterine volume is closely correlated with IVF reproductive outcome in adenomyosis is that, to some extent, the uterine volume represents the accumulation of adenomyosis lesions or the severity of adenomyosis. Adenomyosis itself could cause infertility in a variety of approaches, including an enlarged uterine cavity, dysperistalsis of the uterus resulting in impaired sperm transport [23], the chronic inflammatory cells and inflammatory molecules caused by infiltration of ectopic endometrial glands [9], the increasing estrogen in eutopic endometrium caused by the overexpression of aromatase P450 [24], and alterations of endometrial receptivity-related molecules, such as osteopontin, integrin β3, leukemia-inhibiting factor, and the HOXA-10 gene during the implantation window [7]. Adenomyosis uterine volume increasement indicates the accumulation of lesions and further deterioration of molecular expression and tissue function, which may have a stronger adverse effect on IVF reproductive outcome. Clinically, we found that GnRH-a could improve reproductive outcomes in adenomyosis. Park CW found that GnRH-a pretreatment could reduce uterine volume and increase the clinical pregnancy rate of infertile patients with adenomyosis [7]. Studies by our research team have also shown that GnRH-a pretreatment before FET reduced the miscarriage rate and improved the live birth rate among infertile women with adenomyosis whose uterine volume was 56–100 cm^3^ [25]. However, the optimal GnRH-a treatment cycles in FET and fresh ET cycles need to be further validated in subsequent studies.

In addition to uterine volume, the relationship between other ultrasound indicators of adenomyosis and IVF reproductive outcome deserves to be further explored and analyzed, such as the clinical subtypes (focal/diffuse or internal/external) of adenomyosis. Ultrasound indicators of adenomyosis include the presence of myometrial cysts, fan-shaped echo, hyperechoic islets, globular uterus, thickest/thinnest ratio for uterine wall, maximum width of the junctional zone in the sagittal plane, and irregular appearance of the junctional zone [26–29]. Follow-up studies may further explore the relationship between the above ultrasound indicators or a combination of ultrasound indicators and reproductive outcomes of IVF in adenomyosis to provide evidence to support the best clinical decision.

The strengths of this study were listed as follows. First, this study innovatively identified a turning point of uterine volume (uterine volume at 8 weeks of gestation) for worse IVF reproductive outcomes and found that adenomyosis patients with a uterus larger than 8 weeks of gestation had a higher miscarriage rate and a lower live birth rate. Second, the sample size of this study is large, and a total of 1155 infertile patients with adenomyosis were included, which increase the credibility of the conclusions. The limitation of this study is that it is a retrospective study, which requires further confirmation by subsequent prospective studies.

## Conclusions

IVF reproductive outcome gets worse as uterine volume increases in infertile patients with adenomyosis. Adenomyosis patients with a uterus larger than 8 weeks of gestation had a higher miscarriage rate and a lower live birth rate.

### Supplementary Information

Below is the link to the electronic supplementary material.

Supplementary file1 (DOCX 21 KB)

Supplementary file2 (DOCX 26 KB)

## Data Availability

The data that support the findings of this study are available on request from the corresponding author upon reasonable request.
