# Supplementary material for: Association Between Uterine Volume and In Vitro Fertilization (IVF) Reproductive Outcomes of Infertile Patients with Adenomyosis
Source: Reprod Sci. 2023 May 15;30(10):3123–31. doi: 10.1007/s43032-023-01210-2 (PMC10556154; doi:10.1007/s43032-023-01210-2)
Supplement: Supplementary file 1 — Supplementary file1 (DOCX 21 KB) [file 43032_2023_1210_MOESM1_ESM.docx]

**Supplemental table 1** Baseline characteristics of infertile patients with adenomyosis

| Baseline characteristics, N=1155 | |
| --- | --- |
| Age (years), mean±SD | 34.6±4.3 |
| BMI (kg/m^2^), mean±SD | 23.2±3.8 |
| Infertility type, % |  |
| Primary infertility | 651/1155 (56.4%) |
| Secondary infertility | 504 /1155 (43.6%) |
| Infertility duration (years), median (IQR) | 4.0 (2.0, 7.0) |
| Pregnancy times, median (IQR) | 1.0 (0.0, 2.0) |
| Parity times, median (IQR) | 0.0 (0.0, 0.0) |
| Basal FSH (mIU/ml), median (IQR) | 6.6 (5.2, 8.2) |
| AMH (ng/ml), median (IQR) | 2.0 (1.0, 3.3) |
| Uterine volume (cm^3^), median (IQR) | 89.2 (64.0, 127.5) |
| Uterine volume (cm^3^)(corresponding gestational week), % |  |
| ≤56 (≤4 weeks of gestation) | 210/1155 (18.2%) |
| 56-90 (4-6 weeks of gestation) | 372 /1155 (32.2%) |
| 90-130 (6-8 weeks of gestation) | 309 /1155 (26.8%) |
| 130-180 (8-10 weeks of gestation) | 134 /1155 (11.6%) |
| >180 (>10 weeks of gestation) | 130 /1155 (11.3%) |

BMI, body mass index. SD, standard deviation. IQR, interquartile range.
