# Supplementary material for: Association Between Uterine Volume and In Vitro Fertilization (IVF) Reproductive Outcomes of Infertile Patients with Adenomyosis
Source: Reprod Sci. 2023 May 15;30(10):3123–31. doi: 10.1007/s43032-023-01210-2 (PMC10556154; doi:10.1007/s43032-023-01210-2)
Supplement: Supplementary file 2 — Supplementary file2 (DOCX 26 KB) [file 43032_2023_1210_MOESM2_ESM.docx]

**Supplemental table 2** Baseline characteristics and reproductive outcomes of adenomyosis-associated infertile patients with different uterine volume

|  | N=1155 | | | | |  |
| --- | --- | --- | --- | --- | --- | --- |
| Uterine volume（cm^3^） | ≤56 | 56-90 | 90-130 | 130-180 | ＞180 | P |
| Number | 210 | 372 | 309 | 134 | 130 |  |
| Age (years), mean±SD | 33.1±4.2 | 34.7±4.0 | 35.0±4.5 | 35.2±4.3 | 35.7±4.0 | 0.000 |
| BMI (kg/m^2^), mean±SD | 22.4±3.5 | 23.1±3.7 | 23.2±3.9 | 23.7±3.8 | 23.9±4.0 | 0.002 |
| Infertility type, % |  |  |  |  |  | 0.142 |
| Primary infertility | 108/210 (51.4%) | 214/372 (57.5%) | 174/309 (56.3%) | 76/134 (56.7%) | 79/130 (60.8%) |  |
| Secondary infertility | 102/210 (48.6%) | 158/372 (42.5%) | 135/309 (43.7%) | 58/134 (43.3%) | 51/130 (39.2%) |  |
| Infertility duration (years), median (IQR) | 3.0 (2.0, 6.0) | 3.0 (2.0, 6.0) | 3.0 (2.0, 5.0) | 4.0 (3.0, 5.0) | 4.0 (2.0, 5.0) | 0.186 |
| Pregnancy times, median (IQR) | 0.0 (0.0, 2.0) | 1.0 (0.0, 2.0) | 1.0 (0.0, 2.0) | 1.0 (0.0, 2.0) | 1.0 (0.0, 2.0) | 0.700 |
| Parity times, median (IQR) | 0.0 (0.0, 0.0) | 0.0 (0.0, 0.0) | 0.0 (0.0, 0.0) | 0.0 (0.0, 0.0) | 0.0 (0.0, 0.0) | 0.002 |
| Basal FSH (mIU/ml), median (IQR) | 7.0 (5.8, 8.7) | 6.5 (5.1, 8.4) | 6.6 (5.3, 8.2) | 5.8 (5.0, 8.5) | 6.7 (4.7, 7.8) | 0.107 |
| AMH (ng/ml), median (IQR) | 2.0 (1.0, 3.5) | 2.0 (1.1, 3.2) | 1.7 (0.8, 3.0) | 2.8 (1.7, 4.3) | 1.7 (1.0, 3.7) | 0.131 |
| **First fresh ET cycle, n=943** | | | | | |  |
|  | 182 | 313 | 238 | 109 | 101 |  |
| Clinical pregnancy rate, % | 76/182 (41.8%) | 133/313 (42.5%) | 87/238 (36.6%) | 45/109 (41.3%) | 33/101 (32.7%) | 0.143 |
| Miscarriage rate, % | 17/76 (22.4%) | 37/133 (27.8%) | 15/87 (17.2%) | 14/45 (31.1%) | 14/33 (42.4%) | 0.057 |
| Live birth rate, % | 59/182 (32.4%) | 96/313 (30.7%) | 72/238 (30.3%) | 31/109 (28.4%) | 19/101 (18.8%) | 0.022 |
| **First FET cycle, n=493** | | | | | |  |
|  | 80 | 155 | 125 | 67 | 66 |  |
| Clinical pregnancy rate, % | 37/80 (46.2%) | 61/155 (39.4%) | 43/125 (34.4%) | 33/67 (49.3%) | 25/66 (37.9%) | 0.754 |
| Miscarriage rate, % | 8/37 (21.6 %) | 14/61 (23.0%) | 13/43 (30.2%) | 13/33 (39.4%) | 10/25 (40.0%) | 0.032 |
| Live birth rate, % | 29/80 (36.2%) | 47/155 (30.3%) | 30/125 (24.0%) | 20/67 (29.9%) | 15/66 (22.7%) | 0.100 |
| **Per ET cycle, n=1876** | | | | | |  |
|  | 320 | 599 | 499 | 233 | 225 |  |
| Clinical pregnancy rate, % | 129/320 (40.3%) | 240/599 (40.1%) | 180/499 (36.1%) | 100/233 (42.9%) | 69/225 (30.7%) | 0.076 |
| Miscarriage rate, % | 31/129 (24.0%) | 68/240 (28.3%) | 40/180 (22.2%) | 39/100 (39.0%) | 29/69 (42.0%) | 0.002 |
| Live birth rate, % | 98/320 (30.6%) | 172/599 (28.7%) | 140/499 (28.1%) | 61/233 (26.2%) | 40/225 (17.8%) | 0.001 |

BMI, body mass index. SD, standard deviation. IQR, interquartile range.
